# Topological mapping methods for α-helical bacterial membrane proteins – an update and a guide

**DOI:** 10.1002/mbo3.72

**Published:** 2013-02-14

**Authors:** Salim T Islam, Joseph S Lam

**Affiliations:** Department of Molecular and Cellular Biology, University of GuelphGuelph, Ontario, N1G 2W1, Canada

**Keywords:** C-terminal reporter, deuterium exchange, membrane protein, oxidative labeling, topology determinants, topology mapping

## Abstract

Integral membrane proteins with α-helical transmembrane segments (TMS) are known to play important and diverse roles in prokaryotic cell physiology. The net hydrophobicity of TMS directly corresponds to the observed difficulties in expressing and purifying these proteins, let alone producing sufficient yields for structural studies using two-/three-dimensional (2D/3D) crystallographic or nuclear magnetic resonance methods. To gain insight into the function of these integral membrane proteins, topological mapping has become an important tool to identify exposed and membrane-embedded protein domains. This approach has led to the discovery of protein tracts of functional importance and to the proposition of novel mechanistic hypotheses. In this review, we synthesize the various methods available for topological mapping of α-helical integral membrane proteins to provide investigators with a comprehensive reference for choosing techniques suited to their particular topological queries and available resources.

## Introduction

**Figure 1:**
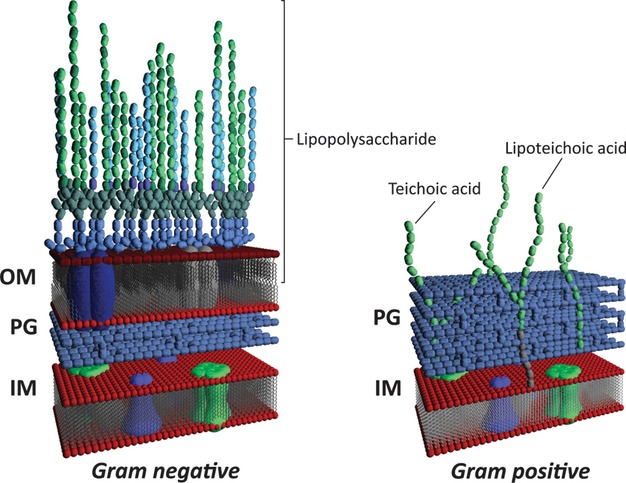
Cell envelope architecture in Gram-positive and Gram-negative bacteria. Membrane-spanning proteins are present in the inner membrane of both types of bacteria, and are also present in the outer membrane of the latter. IM, inner membrane; PG, peptidoglycan; OM, outer membrane. Figure courtesy of Dr. Wayne L. Miller.

Bacterial membrane proteins are responsible for a wide range of cellular processes such as energy production (D'Alessandro and Melandri 2010), substrate import/export (Islam and Lam 2013), signal transduction (Sourjik and Wingreen 2012), motility (Patrick and Kearns 2012; Zhang et al. 2012), and virulence factor production/secretion (Tseng et al. 2009; Lam et al. 2011). Although Gram-negative and Gram-positive organisms have different cell envelope architectures, the cytoplasmic membrane in each shares common properties and is the site at which many of the important processes above occur due to the functions of membrane proteins (Fig. 1).

Although membrane proteins are physiologically important and represent 25% of all proteins identified, the proportion of data on their tertiary structures is vastly underrepresented in the Protein Data Bank (White 2009), with only 359 unique membrane protein structures deposited to date among the >85,000 entries (White Laboratory [http://blanco.biomol.uci.edu/mpstruc/listAll/list]). This is largely due to inherent difficulties in overexpression, purification, and manipulation of these proteins. To advance our understanding of the structure and function of membrane proteins, investigators have taken to experimentally mapping their topologies. In recent years, a surge of publications concerning the topological mapping of integral inner membrane (IM) bacterial proteins has appeared in the literature, with a wide range of techniques having been employed. Within the scope of this review, we have synthesized and compared the various experimental topological mapping methodologies available to researchers working with membrane proteins such that appropriate techniques and principles can be chosen to suit the particular hypotheses being investigated.

## Determinants of Membrane Protein Topology

Insertion of proteins into the IM of Gram-negative and Gram-positive bacteria (Fig. 1) is governed by multiple trafficking pathways (Dalbey and Kuhn 2012), with three mechanisms in particular playing prominent roles. The majority of IM proteins require the Sec translocase for membrane insertion through the cotranslational targeting of ribosome–nascent peptide chain complexes (Driessen and Nouwen 2008; Xie and Dalbey 2008); this pathway is able to mediate insertion of multi-transmembrane segment (multi-TMS) proteins with a range of periplasmic and cytoplasmic loop characteristics. The second pathway involves the action of the YidC insertase, which can independently mediate IM insertion of certain membrane proteins; these proteins typically possess one or two TMS connected by short translocated loops (Xie and Dalbey 2008). The basis for “YidC-associated” specificity remains poorly understood, but substrates identified to date are mainly components of large oligomeric complexes, suggesting that YidC is involved in the assembly of multimeric assemblies (Kol et al. 2008). YidC can also function in concert with the Sec translocon to facilitate insertion of a subset of membrane proteins, indicating association between these systems (Dalbey et al. 2011). Prefolded proteins can also be translocated across the IM via the twin-arginine translocation (Tat) machinery. Several Tat substrates have been demonstrated to be membrane bound through the presence of a single TMS at either the N- or the C-terminus of the protein, indicating that the Tat pathway can also mediate successful IM insertion of select proteins (Palmer and Berks 2012). As the molecular mechanisms of these insertion systems are quite complex, they are beyond the scope of this review. However, more detailed information can be found in the comprehensive review articles cited above. Nonetheless, during the biosynthesis and insertion processes, certain properties of TMS and loop sequences have been found to govern the overall topogenesis of IM proteins to facilitate their placement and retention in the membrane.

### Charged residues

**Figure 2:**
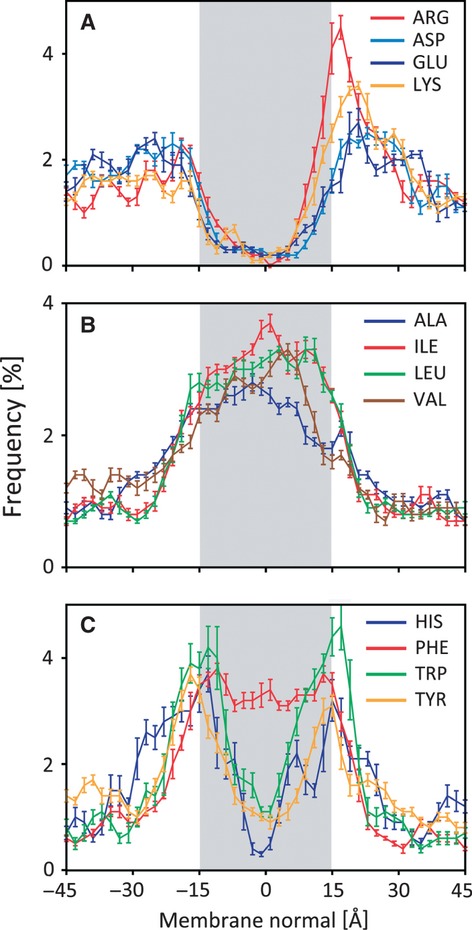
Amino acid distributions in existing membrane protein structures. (A) Charged residues. (B) Hydrophobic residues. (C) Aromatic residues. Regions in gray represent membrane-spanning transmembrane segments (TMS). Figure modified from Ulmschneider et al. (2005).

The significance of charged residues in conferring final topological character to IM proteins is indisputable, with numerous investigations illustrating the importance of charge disposition on the placement, orientation, and delimitation of TMS and loop domains (Nilsson and von Heijne 1990; Gafvelin and von Heijne 1994; Seppälä et al. 2010). Much of the importance has been attributed to the “positive-inside rule,” which prescribes that the cytoplasmic face of membrane proteins possesses distinctly positive charge character compared with the periplasmic face due to an overrepresentation of cationic amino acids in the former. This postulate was initially proposed based on the examination of various published topological investigations (von Heijne 1986) and subsequently reinforced through the comparative analysis of residue distributions in existing biophysical membrane protein structures. Findings from the latter study revealed that while negatively charged Asp and Glu residues are distributed evenly between periplasmic and cytoplasmic loops, positively charged Arg and Lys residues are most frequently located on the cytoplasmic face of existing membrane protein structures (Fig. 2A), while hydrophobic residues are expectedly distributed throughout the membrane (Fig. 2B) (Ulmschneider et al. 2005). However, a common misconception is that this “rule” is an absolute requirement, when in reality, cytoplasmic loops with net anionic character have been identified (Allard and Bertrand 1992; Pi et al. 2002; Zhang et al. 2003, 2005). Furthermore, the charges have a general density-dependent additive effect, with negligible importance associated with their exact primary structure locations within the various loops (Andersson et al. 1992).

However, in situations where anionic amino acids substantially outnumbered cationic residues in a given domain, the former have been shown to promote domain translocation (Elofsson and Heijne 2007). This potential to affect domain translocation has also been demonstrated in instances of anionic residues within six positions of the TMS-loop interface (Rutz et al. 1999) as well as in an instance of a TMS with minimal hydrophobicity (Delgado-Partin and Dalbey 1998). Ultimately, the reason behind the predominance of positively charged amino acids as membrane-retention signals for TMS over those that are negatively charged is not yet understood. The relevance of membrane interface charge character for a given membrane protein is also such that the local charge of the membrane environment, conferred by lipid headgroups, can affect folding and hence topology (DeChavigny et al. 1991; van Klompenburg et al. 1997; Bogdanov et al. 2002), while still satisfying the “positive-inside rule” (Bogdanov et al. 2009). However, the role of lipids in membrane protein topogenesis has recently been comprehensively reviewed elsewhere (Dowhan and Bogdanov 2009) and as such will not be discussed herein.

### Aromatic residues

Amino acids with aromatic functional groups play an important role in delineating the boundaries of TMS (de Planque et al. 2002), likely through the ability to interact with both the polar and apolar interface regions. The hydrophobicity of the planar ring structure confers the potential to interact with lipid acyl chains, while the presence of amide groups confers the ability to undergo hydrogen-bonding interactions (Ippolito et al. 1990) at the polar interface zone. Incidentally, the aromatic amino acids Trp, Tyr, and His have a very high frequency of localization at TMS-membrane interface junctures in existing membrane protein structures (Fig. 2C) (Ulmschneider et al. 2005). The presence of hydrogen-bonding side chains for these amino acids embedded deep within a hydrophobic membrane bilayer environment would almost certainly confer an entropic disadvantage, accounting for the enrichment of these three amino acids at interfacial regions. Further support for this proposition is derived from the observed unbiased distribution of hydrophobic Phe residues throughout membrane-spanning domains of membrane proteins (Fig. 2C) (Ulmschneider et al. 2005), with the only chemical difference between Phe and Tyr side chains being a lack of a hydroxyl group in the former.

## Topological Mapping Approaches

Topological mapping is an important tool to identify protein domains that are exposed or embedded in the membrane; these data can be invaluable for characterizing membrane proteins for which high-resolution structural studies are not feasible. In turn, topological mapping has led to the discovery of amino acid tracts of structural and catalytic importance as well as to the development and refinement of novel mechanistic hypotheses. Various methods have been used to map the topology of integral membrane proteins, each with its inherent benefits and caveats. The choice of reporter methodology employed could depend on a multitude of factors, including access to specific equipment, cost of sample processing, rapidity of obtaining results, and most importantly, the precise question to which topological answers will be of benefit.

### C-terminal reporter fusions

The use of C-terminal fusions to various reporter proteins is a widely used method for mapping membrane topology. It involves genetically fusing reporter genes to various 3′ truncations of the gene of interest (Table 1). Translated truncation products result in the localization of the fused C-terminal reporter constructs to different subcellular compartments, directly promoting or inhibiting activity of a given reporter. Activity of the reporter (or lack thereof) can be directly assayed by visually examining colony phenotypes as a preliminary screen, followed by quantitatively assaying for reporter activity to yield concrete results for the subcellular localization of a particular truncation residue.

**Table 1:** C-terminal reporter fusion tags

Localization	Reporter	Detection substrates/Conditions	Phenotype	References
Periplasm	Bla	β-lactam antibiotics (e.g., ampicillin)	• Resistance to β-lactam antibiotics	Broome-Smith et al. (1990)
	PhoA	BCIP (in vivo), *p*NPP (in vitro)	• BCIP hydrolysis turns colonies blue	Manoil and Alan (1991)
			• *p*NPP hydrolysis turns buffer yellow (*A*_420_)	
	scFv	Fluorescent hapten digoxin-BODIPY	• Fluorescence emission	Jeong et al. (2004)
Cytoplasm	CAT	Chloramphenicol	• Resistance to chloramphenicol	Zelazny and Bibi (1996)
	LacZ	X-gal (in vivo), ONPG (in vitro)	• X-gal hydrolysis turns colonies blue	Manoil and Alan (1991)
			• ONPG hydrolysis turns buffer yellow (*A*_420_)	
	GFP	Excitation at 395 nm (or 475 nm)	• Fluorescence emission at 509 nm	Drew et al. (2002)
Periplasm, cytoplasm, TMS	PhoA-LacZα	BCIP and Red-Gal (in vivo), *p*NPP and ONPG (in vitro)	• BCIP hydrolysis turns colonies blue	Alexeyev and Winkler (1999)
			• Red-Gal hydrolysis turns colonies red	
			• Simultaneous BCIP and Red-Gal hydrolysis turns colonies purple	
			• *p*NPP and ONPG hydrolysis turns buffer yellow (*A*_420_)	

Bla, β-lactamase; PhoA, alkaline phosphatase; BCIP, 5-bromo-4-chloro-3-indolyl phosphate; *p*NPP, *p*-nitrophenyl phosphate; scFv, single-chain antibody variable region; CAT, chloramphenicol acetyltransferase; LacZ, β-galactosidase; X-gal, 5-bromo-4-chloro-3-indolyl-β-d-galactoside; ONPG, *o*-nitrophenyl-β-galactoside; GFP, green fluorescent protein.

The C-terminal reporter approach does not depend on full-length protein analysis; this precludes the need for the overexpression and purification of membrane proteins for which these processes are notoriously difficult. Instead, the use of C-terminal reporter fusions relies on the formation of secondary structure by a membrane protein, particularly that of TMS, during cotranslation. As the α-helical TMS are already folded upon entry into the IM, any intervening cytoplasmic or periplasmic loop is now localized to its respective subcellular region, along with any fused reporter moiety, a process largely independent of tertiary structure packing events between translated TMS (Dowhan and Bogdanov 2009). For this reason, C-terminally truncated proteins fused to various reporter tags can be exploited to map the topology of a given membrane protein, provided that sufficient truncation coverage across the entire protein has been obtained. Additionally, such techniques can yield representative approximations of the topology seen from end-stage X-ray crystal structures (Cassel et al. 2008). However, for certain membrane proteins, the proper membrane insertion of α-helical TMS of marginal hydrophobicity has been shown to be affected by the presence of a particular upstream or downstream TMS, suggesting that local sequence context can be important for the membrane insertion of certain marginally hydrophobic TMS (Hedin et al. 2010). C-terminally truncated proteins fused to a reporter may be inactive, depending on the extent of the truncation; however, this characteristic may prove useful if one is interested in examining the minimum length of a protein required for function. Notwithstanding, the tractability, ease of use, reproducibility, and rapid screening afforded by the use of C-terminal reporter fusions (Table 1) make it an effective tool for mapping the topology of α-helical integral membrane proteins.

#### Periplasmic reporters

To avoid conflicting results, proteins deemed suitable for use as periplasmic reporters should ideally be active only in the periplasm, and not in the cytoplasm. Initially, the fusion of β-lactamase to predicted periplasmic portions of a membrane protein was used to confer resistance to β-lactam antibiotics (Broome-Smith et al. 1990). As the targets of these drugs are the enzymes responsible for cell wall biosynthesis, the presence of β-lactamase in the cytoplasm would render the particular cells susceptible to these drugs, thus selecting for periplasmic fusions. However, the use of β-lactamase fusions has become supplanted with the creation of fusions to alkaline phosphatase (PhoA), a zinc metalloprotein that only forms the disulfide bonds required for proper folding once exported to the periplasm (Manoil et al. 1990). Fusions to PhoA are the most widespread periplasmic reporter fusions. Colonies expressing periplasmic PhoA fusions can be visually screened by supplementation of the agar medium with a PhoA-specific substrate such as 5-bromo-4-chloro-3-indolyl phosphate (BCIP), yielding pigmented (blue) colonies. Quantitative values for enzyme activity can also be obtained in vitro by measuring the breakdown of the chromogenic substrate *p*-nitrophenyl phosphate (*p*NPP) (Manoil and Alan 1991).

A periplasm-specific fluorescent reporter system has also been developed in which an anti-hapten single-chain antibody variable region fragment (scFv) peptide is encoded as a fusion to a domain of interest. The 26-10 scFv binds with high specificity and affinity to the haptens digoxigenin and digoxin (Chen et al. 1999). Labeling of scFv fragments expressed in the periplasm is accomplished through permeabilization of the outer membrane in the presence of digoxin conjugated to the fluorescent dye BODIPY (Jeong et al. 2004). Expression of cytoplasmically located scFv fragments will not result in labeling as digoxin-BODIPY cannot cross the IM. Furthermore, proper folding of scFv peptides requires disulfide bond formation, which is not favored in the reducing environment of the cytoplasm (Levy et al. 2001). Together, this system allows for selective fluorescent labeling of periplasmic domains and downstream sorting via flow cytometry (Jeong et al. 2004).

#### Cytoplasmic reporters

Antibiotic resistance has also been used as a marker of cytoplasmic reporter localization, with chloramphenicol acetyltransferase used for this purpose. Due to the requirement of cytoplasmic acetyl coenzyme A for the inactivation of chloramphenicol via this mechanism, periplasmic fusions can be selected against (Zelazny and Bibi 1996). However, as with periplasmic fusions, antibiotic selection has been largely abandoned in favor of cytoplasmic reporters that will yield distinguishable colony phenotypes. The most popular cytoplasmic reporter protein has been β-galactosidase (LacZ), which must form a tetramer in the cytoplasm to be active (Matthews 2005). Fusion of LacZ to cytoplasmic residues results in high LacZ activity, which can be easily visualized through breakdown of 5-bromo-4-chloro-3-indolyl-β-d-galactoside (X-gal) or related substrates on agar plates, yielding colony pigmentation. LacZ activity in fusion proteins can be readily quantified through measuring in vitro breakdown of the chromogenic substrate *ortho*-nitrophenyl-β-galactoside (ONPG) (Manoil and Alan 1991). However, fusion of full-length LacZ to periplasmic domains may still yield low levels of reporter activity (Froshauer et al. 1988) and can often lead to toxicity (Lee et al. 1989).

More recently, green fluorescent protein (GFP) has been employed as a cytoplasmic reporter for topology mapping studies (Drew et al. 2002). This approach is based on the inability of GFP to fluoresce in the periplasm when expressed as a fusion to a cotranslationally IM-inserted protein (Feilmeier et al. 2000). The inability to fluoresce is likely due to misfolding of the GFP following Sec-dependent extrusion, as a prefolded GFP-fusion protein exported to the periplasm via the Tat pathway (De Buck et al. 2008), has been shown to remain fully active and fluorescent (Thomas et al. 2001). This misfolding is attributed to the exposure of Cys residues 49 and 71 during folding in an oxidizing environment such as the periplasm in Gram-negative bacteria or the lumen of the endoplasmic reticulum in eukaryotic cells, likely resulting in the binding of Cys-containing proteins or other folding GFP molecules (Aronson et al. 2011). Upon correct folding of GFP, these two Cys residues flank the Ser65-Tyr66-Gly67 chromophore of GFP in the β-barrel interior, but are spaced far enough apart that they do not form an intramolecular disulfide bond (Ormö et al. 1996; Reid and Flynn 1997). Strong evidence exists for the role of Cys residues in the lack of fluorescence observed following cotranslational IM insertion (Feilmeier et al. 2000); this is further supported by the ability of the fluorescent protein mCherry, which lacks native Cys residues, to undergo proper folding and emit fluorescence when expressed in the periplasm (Chen et al. 2005; Aronson et al. 2011).

Recently, a highly efficient and stable folding variant of GFP, termed “superfolder GFP” (sfGFP) (Pédelacq et al. 2006), in conjunction with an optimized signal sequence (Lee and Bernstein 2001), was demonstrated to result in Sec translocon-targeted sfGFP expression and fluorescence in the periplasm (Aronson et al. 2011). As two of the sfGFP substitutions (S30R and Y39N) occur upstream of the two Cys residues described above (Pédelacq et al. 2006), it is highly likely that sfGFP is able to form a folding intermediate in the periplasm that shields the Cys residues from disulfide bond formation with other components. Given the periplasmic fluorescence capabilities of sfGFP, it may prove to be a useful reporter for periplasmically expressed proteins.

Alternatively, sfGFP has been evolved via DNA shuffling (Stemmer 1994) to serve as a cytoplasmic reporter through use of the self-assembling split GFP (saGFP) approach, which involves separate expression of β-strands 1–10 ([1–10_OPT_]) and modified β-strand 11 ([11_H7_]) of the GFP molecule. When expressed in the same compartment, [1–10_OPT_] and [11_H7_] are able to self-assemble and produce a functional fluorophore (Cabantous et al. 2005; Toddo et al. 2012). This system had been previously used to determine the orientation of full-length multi-TMS proteins (containing a C-terminal fusion to modified β-strand 11) in plastids of *Toxoplasma gondii* (van Dooren et al. 2008) and *Arabidopsis thaliana* (Sommer et al. 2011). Recently, [11_H7_] was fused to the N-terminus of single-TMS IM proteins in *Escherichia coli*; expression of the [11_H7_] fusion constructs followed by cytoplasmic expression of [1–10_OPT_] resulted in whole-cell fluorescence for proteins with cytoplasmic N-termini. Conversely, saGFP was not found to reassemble in the periplasm, resulting in an absence of fluorescence. As the [11_H7_] peptide is only 18 amino acids in length, it represents a useful reporter motif for both the N- and C-termini of membrane proteins as it should minimize the perturbation of secondary and tertiary structure. However, a limitation of saGFP for topology mapping purposes is its requirement for cytoplasmic localization, as saGFP was not found to reassemble in the periplasm, resulting in an absence of fluorescence in this compartment (Toddo et al. 2012).

Due to the large selection of GFP variants now at the disposal of investigators, special consideration should be taken to describe the exact variant of GFP employed in topology mapping studies as different variants can confer different properties. However, these varying characteristics can be taken advantage of as long as investigators understand the fundamental differences between the various modifications that have been introduced to the original GFP molecule.

#### Dual reporters

The standard approach to obtaining localization data for a particular amino acid via C-terminal fusions is to create separate fusions to a periplasmic and a cytoplasmic reporter, followed by quantitation of reporter activity. In this manner, relative (instead of absolute) enzyme activities can be determined and used for comparison between different residues. However, the level of expression of the protein of interest can vary depending on the respective periplasmic or cytoplasmic reporter tag. This can lead to difficulties in normalization of the data.

To circumvent the aforementioned concerns of individual reporter usage as well as the need to normalize data between different fusions, Alexeyev and Winkler (1999) created a chimeric PhoA-LacZα reporter, which encodes full-length PhoA fused to the alpha fragment of LacZ. When expressed in the periplasm, this dual reporter exhibits high alkaline phosphatase activity, with no cytotoxic effects exhibited by the presence of the attached LacZα fragment (unlike with full-length LacZ). Conversely, cytoplasmic localization of the reporter only displays high β-galactosidase activity, due to complementation of the reporter LacZα fragment with a chromosomally encoded LacZω fragment to reconstitute functional LacZ in the cytoplasm (Alexeyev and Winkler 1999). As such, the capability for two different enzyme activities in a single reporter construct bypasses the need for separate fusions. Furthermore, supplementation of fusion library transformation recovery agar plates with both PhoA- and LacZ-specific chromogenic substrates results in visually distinguishable colony color phenotypes that correlate with the localization of the dual reporter in a given construct. Therefore, this chimeric PhoA-LacZα reporter system is designed to facilitate rapid preliminary screens for cytoplasmic, transmembrane, and periplasmic residue localizations.

Although the level of fusion protein expression for different residues may affect the absolute activities of each enzyme, the relative ratio of activities of the two enzyme components would not be affected for a specific amino acid. Hence, PhoA and LacZ activities for each fusion can be normalized to the highest activity recorded for each reporter within the fusion library to obtain a normalized activity ratio. The merit of determining the ratio of PhoA:LacZ activities is that it allows for the direct comparison of localization data between all residues screened within a specific protein (Alexeyev and Winkler 1999), as well as between multiple proteins from a single physiological pathway (Islam et al. 2010).

#### Sandwich fusions

The various enzyme reporter fusions described above have been presented within the context of a C-terminally truncated construct of the protein undergoing topological mapping; as such, the downstream polypeptide corresponding to the remainder of the protein of interest is not present as it has not been translated. To supplement these data, reporter protein constructs lacking a stop codon can be genetically inserted within the coding sequence for a membrane protein such that the reporter construct is in frame with both the upstream and downstream portions of the gene (Doi and Yanagawa 1999). The resultant translated product, termed a “sandwich fusion,” now contains the wild-type amino acid sequence upstream of the insertion, allowing the protein to fold and pack efficiently. This is followed by the in-frame translated reporter moiety, then the remainder of the amino acid sequence of the target protein. Random insertion of the reporter moiety is typically mediated by transposon integration (Ehrmann et al. 1990; Mealer et al. 2008), while targeted insertion can be accomplished through an approach such as gene splicing by overlap extension (Horton 1995).

Sandwich fusions that maintain near-native TMS packing are typically those that are expressed in large loop domains, allowing for the proper folding of the reporter construct such that perturbations to the insertion of downstream membrane protein TMS are minimized (Doi and Yanagawa 1999). The activity of the particular reporter is assayed in the same manner described above (Alexeyev and Winkler 1999). These characterizations can help to understand the positioning of buried TMS that are less hydrophobic and which may require the presence of upstream TMS in the protein to properly fold and pack. However, proximity of the N- and C-termini of the inserted reporter are required to ensure that downstream target protein-specific TMS are able to properly insert into the membrane; for this reason, sandwich fusions in TMS or short loops can be disruptive to the tertiary structure of a protein with multiple TMS.

### Site-specific label detection

The specific side chain chemistry of various amino acids can be used to covalently label various solvent-exposed residues. These labels can in turn be detected through a range of biochemical and/or biophysical techniques (Table 2). The side chains of native residues can be detected via this technique. Alternatively, nonnative residues can be introduced for the purpose of detection with these probes. However, the functionality of these substituted mutants must also be examined to determine their ability to maintain protein function, potentially identifying catalytically important residues (Frillingos et al. 1998); if such substitutions are found to affect function, a more innocuous amino acid substitution such as Ala should be introduced to confirm its importance.

**Table 2:** Site-specific label probes

Probe	Amino acid target	Detection method	Phenotype	References
MPB	Cys	Western immunoblot	• Biotin tag detected with streptavidin	Bogdanov et al. (2005)
OGM	Cys	In-gel fluorescence (Excitation at 496 nm)	• Fluorescence emission at 524 nm	Culham et al. (2003)
^•^OH	Met, Cys	Liquid chromatography, mass spectrometry	• Met-containing peptides increase by +16 Da	Konermann et al. (2011)
			• Cys-containing peptides increase by +48 Da	
DiPC	Glu, Asp	Liquid chromatography, mass spectrometry	• Glu and Asp-containing peptides increase by +126 Da	Weinglass et al. (2003)

MPB, *N*-(3-maleimidyl-propionyl)biocytin; OGM, Oregon green 488 carboxylic acid; ^•^OH, hydroxyl radical; DiPC, diisopropylcarbodiimide.

#### Substituted-cysteine accessibility method

Substituted-cysteine accessibility method (SCAM) involves working directly with functional variants of a protein of interest and takes advantage of the unique side chain chemistry of Cys residues (Liapakis et al. 2001; Bogdanov et al. 2005). The process first requires mutagenesis of the protein of interest to substitute all native Cys residues, usually with Ser or Ala, contingent on the Cys residues not being required for protein function (Liapakis et al. 2001). This step has the potential to affect the native function of a given protein through the destruction of required Cys bridges (Sur et al. 1997; Köhler et al. 2003), changes to the natural oligomeric state (Kao et al. 2008), or alteration of protein stability or membrane trafficking (Pajor et al. 1999). Targeted Cys substitutions are subsequently introduced at various positions throughout the primary amino acid sequence of the protein (Liapakis et al. 2001). The degree of Cys substitution within the target protein directly affects the quality of the final topological model, with poor resolution obtained through use of only a few substitutions (Lu et al. 2007), while higher resolution can be attained by more extensive coverage of site-specific substitutions (Frillingos et al. 1998; Bogdanov et al. 2005).

Following expression of Cys-substituted mutants, in vivo labeling with a thiol-reactive reagent is carried out. Biotin-linked *N*-(3-maleimidylpropionyl)biocytin (MPB) or UV-excitable Oregon green 488 maleimide carboxylic acid (OGM) are commonly used for this purpose due to their low membrane permeability and ability to form stable bonds with thiol groups depending on the availability of a water molecule in the local environment.

For MPB-labeled proteins, they are subsequently solubilized, affinity purified, resolved via SDS-PAGE (sodium dodecyl sulfate polyacrylamide gel electrophoresis), and blotted to a nitrocellulose membrane, after which the biotin label is detected with streptavidin-linked detection reagents (Bogdanov et al. 2005). In this manner, only water-accessible Cys-substituted periplasmic residues would be detected. However, prior treatment with the blocking agent (2-[trimethylammonium]ethyl)methanethiosulfonate bromide (MTSET) can prevent labeling of Cys substitutions with MPB. MTSET is a thiol-specific reagent similar to MPB in its reactivity; however, it is charged and cannot pass through the IM. Therefore, periplasmic localization can be confirmed for residues that can be initially labeled with MPB, but for which this phenotype is absent with prior MTSET treatment. Conversely, cytoplasmic residues can also be labeled with MBP; however, this requires much higher concentrations of the labeling reagent in addition to extended incubation times. Individual Cys-substituted constructs can also be purified and reconstituted in membrane vesicles in which the periplasmic and cytoplasmic faces of the protein can adopt both a vesicle lumen-facing and a vesicle exterior-facing orientation, allowing for simultaneous MPB labeling of accessible periplasmic and cytoplasmic residues (Bogdanov et al. 2005).

For OGM-labeled bacterial cell samples, in addition to initial surface labeling (OGM^+^/MTSET^−^), an identical second aliquot is treated only with MTSET, while a third is left untreated. Cells in all three aliquots are then lysed, with the membranes in the second (OGM^−^/MTSET^+^) and third (OGM^−^/MTSET^−^) preparations subsequently treated with OGM. This approach yields cell samples with only periplasmic labeling (aliquot 1), only cytoplasmic labeling (aliquot 2), or simultaneous periplasmic and cytoplasmic labeling (aliquot 3). Each of the three sets of proteins is affinity purified and immediately resolved via SDS-PAGE, followed by exposure to UV light to detect in-gel OGM fluorescence, thus indicating the presence/absence of labeling (Culham et al. 2003). This step avoids the requirement for Western blotting (during MPB labeling), which is notoriously inefficient for membrane proteins (Abeyrathne and Lam 2007).

Although information via SCAM is obtained directly at the protein level, different expression levels of various Cys-substituted protein variants can occur. Furthermore, thiol-labeling rates can vary, depending on the subcellular localization of Cys-substituted residues (Liapakis et al. 2001; Bogdanov et al. 2005). Finally, certain maleimide reagents are not completely specific to sulfhydryl functional groups, as stable, efficient, and preferential modification of lysine side chains over cysteine side chains has been observed at pH 7.3 (Holbrook and Jeckel 1969). Nonetheless, SCAM is a powerful, well-tested, and popular technique for examining membrane protein topology.

#### Oxidative labeling

Solvent-accessible side chains can also be labeled using hydroxyl radicals (^•^OH) (Takamoto and Chance 2006), which can be easily generated using a pulsed UV laser to photolyze dilute H_2_O_2_ (Hambly and Gross 2005). This technique has recently come to the forefront of examining topology for membrane proteins in vitro both in their natural lipid background (Pan et al. 2008) and solubilized in detergent micelles (Pan et al. 2012). Following ^•^OH labeling of side chains, the membrane protein of interest is digested with site-specific proteases such as trypsin, after which proteolytic peptides are analyzed via liquid chromatography (LC)-mass spectrometry (MS). However, the proteolysis and subsequent fragmentation steps may be difficult for short-looped proteins for which specific protease recognition sites may not be accessible. Successful ^•^OH labeling typically results in +16 Da increases in molecular mass corresponding to the addition of a single oxygen atom, although higher-integer multiples (e.g., +32 Da, +48 Da) are possible for certain residues (Takamoto and Chance 2006). Both Met and Cys residues react readily with ^•^OH due to their side chain sulfur atoms, with Met typically increasing by +16 Da; Cys mainly increases by +48 Da, forming anionic cysteinic acid that is not conducive to detection via MS in positive-ion mode (Takamoto and Chance 2006). When compared with proteolytic peptides from unlabeled preparations, LC-MS detection of modified peptides can be used to examine the solvent accessibility of periplasmic and cytoplasmic loops in membrane proteins, as well as those potentially contacting an interior lumen in channel-forming proteins. Through an analogous approach, the solvent accessibility of the carboxyl-containing side chains of Glu and Asp can be probed with the use of the hydrophobic carbodiimide diisopropylcarbodiimide (DiPC). Residue modification with DiPC results in a +126 Da increase in molecular mass, which can also be detected through comparison of fragmented peptides from labeled versus unlabeled proteins (Weinglass et al. 2003).

### Deuterium exchange-mass spectrometry

**Figure 3:**
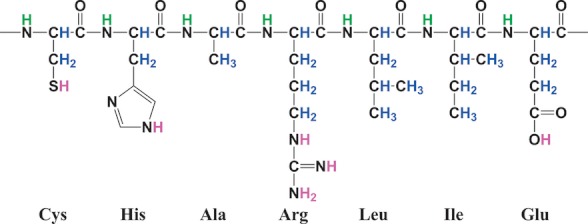
Different environments for hydrogen atoms in a protein. *Magenta*, side-chain protons that exchange rapidly with solvent. *Blue*, carbon-bound protons that are strongly bonded and rarely exchange with solvent. *Green*, amide protons that exchange with solvent at measurable rates.

It has been known for over 50 years that certain protons (H^+^) within a native protein will exchange more readily than others in water (Lenormant and Blout 1953). This principle has been developed into a powerful technique in which solvent-accessible protein domains can be labeled with deuterium and subsequently detected via MS through analysis of +1 Da increases in peptide mass (Percy et al. 2012). Hydrogen is present in three interaction settings within proteins. The first involves those covalently attached to carbon; whether present on the backbone or on side chains, these are stably bonded and do not readily exchange with solvent. Conversely, side chain hydrogen atoms bound to nitrogen, oxygen, or sulfur cannot be detected as they exchange too quickly. However, amide hydrogens in the protein backbone undergo detectable rates of exchange with the solvent, allowing for deuterium exchange-MS (DX-MS) to be carried out (Fig. 3); these atoms also take part in hydrogen-bonding interactions in α-helical and β-sheet secondary structures, as well as tertiary structure packing events, each of which affects their rate of exchange (Englander et al. 1972). For integral membrane proteins, domains accessible for DX-MS would constitute those exposed in periplasmic or cytoplasmic loops (as well as those in potential channel lumens) (Englander et al. 1996) making this an ideal technique with which to examine membrane topology. By extension, through differential labeling rates for various backbone positions, dynamic protein regions can be detected through extended periods of deuterium incubation, providing additional functional insights (Zhang et al. 2010).

**Figure 4:**
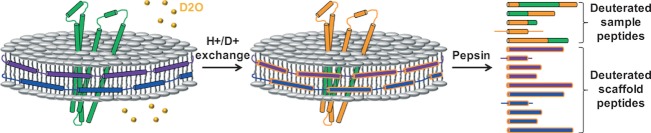
Nanodisc-assisted deuterium exchange workflow. Nanodisc-incorporated membrane proteins are bathed in deuterated water (D_2_O), resulting in amide-position D^+^ labeling of the protein for accessible/exposed sites. Nanodisc disassembly and pepsin digestion are followed by ultra performance liquid chromatography (UPLC) to separate peptides and mass spectrometry (MS) to identify deuterated fragments.

Recently, an exciting advancement in the use of DX-MS for topological mapping has come about through the incorporation of Nanodisc technology (Hebling et al. 2010). Nanodiscs are soluble self-assembling nanoscale phospholipid bilayers, encircled by two copies of a membrane scaffold protein, in which single integral membrane proteins can be reconstituted in order to better mimic their native lipid environment compared with detergent micelles (Bayburt and Sligar 2010). To map topology using this setup, nanodisc-reconstituted proteins are subjected to deuterium exchange at pH 7.0 for different durations, allowing for labeling of exposed protein domains on both sides of the Nanodisc. The labeling reaction is quenched by reducing the pH to 2.5, followed by addition of cholate to disassemble the Nanodisc scaffold. The deuterated protein is subsequently digested with pepsin, which is robust and retains proteolytic activity at pH 2.5. Lipids are abstracted from the mixture through addition of zirconium oxide beads, after which ultra performance liquid chromatography (UPLC) is used to separate peptic membrane protein peptides from peptides derived from the scaffold proteins. The resolved ions are then analyzed by electrospray ionization (ESI)-MS to identify deuterated peptides, from which labeling rates can be determined, allowing for the solvent-accessible regions of the protein to be elucidated (Fig. 4) (Hebling et al. 2010). However, the potential masking of sample-derived peptide signals by those corresponding to background scaffold protein fragments remains a drawback of this method as signal footprints from the latter peptides must be discounted to properly analyze only those corresponding to the protein of interest (Morgan et al. 2011). Furthermore, successful proteolytic digestion is required for this method, which may be a limiting factor for certain proteins. This is an important step in the procedure as both sequence coverage and spatial resolution of DX-MS can be improved through the generation of short overlapping peptides (Hoofnagle et al. 2003; Ahn et al. 2013).

### Reporter-fusion topology mapping methodology

#### In silico TMS prediction consensus

**Figure 5:**
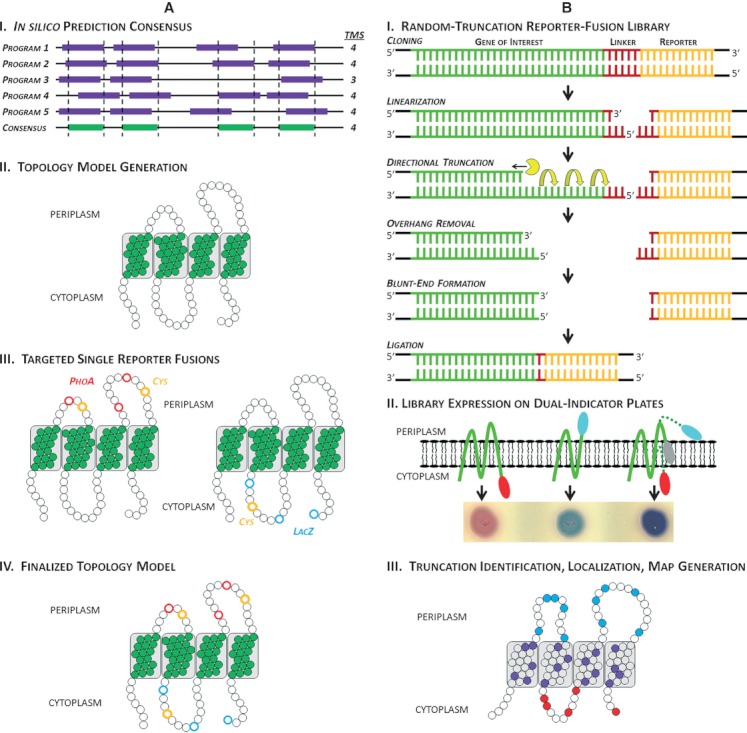
Approaches to determining reporter/site-specific label localization. (A) In silico-based method for deciding reporter position. (I) The amino acid sequence of interest is subjected to topology prediction analysis by several prediction algorithms, with the location of transmembrane segments (TMS) within the protein specified by consensus positions between the various algorithms. (II) Based on consensus TMS localization, a topology model is generated. (III) Targeted fusion/label sites are created for loop positions, with separate periplasmic and cytoplasmic reporter constructs for enzyme fusions. (IV) The enzyme activity of the respective fusions or the detection of a site-specific label is assayed to validate the in silico-based model. (B) Randomized method for obtaining random reporter fusion positions. (I) The gene of interest is cloned upstream of the reporter construct (*phoA-lacZα* reporter used as a representative reporter), after which the fusion construct is linearized between the gene and reporter. Processive digestion via exonuclease III creates 3′ gene truncations; as the digestion reaction continues, aliquots are removed at regular intervals and pooled in a common “stop” tube to yield a pool of randomly 3′-truncated gene constructs; 5′ overhangs from the common pool are removed via treatment with mung bean nuclease, after which blunt ends are introduced through treatment with the Klenow fragment of DNA polymerase I. Finally, the truncated genes are fused to the reporter construct via blunt-ended ligation. (II) Translation of various truncated constructs in frame with the reporter results in localization of the PhoA-LacZα reporter in the cytoplasm, periplasm, or within the membrane, yielding different ratios of enzyme activities. These different ratios result in different relative amounts of PhoA-specific (BCIP [5-bromo-4-chloro-3-indolyl phosphate]) and LacZ-specific (RedGal) substrate breakdown, causing blue and red colony pigmentation for periplasmic and cytoplasmic truncations, respectively. For TMS-localized fusions, the constructs adopt a so-called “frustrated” topology wherein a certain proportion display the reporter in the periplasm, while the remainder display the reporter in the cytoplasm; thus, for a single TMS-based fusion, both periplasmic and cytoplasmic reporter enzyme activities are produced in the same cell, resulting in simultaneous production of blue and red pigmentation to produce purple colony coloration. Depending on the construct, the proximity of a fusion to the periplasm or cytoplasm can sometimes be qualitatively evaluated based on the degree of blue-shifted or red-shifted purple pigmentation. (III) Constructs from pigmented colonies are sequenced to identify the position of 3′ gene truncation and reporter fusion. Normalized enzyme activity ratios for the various fusions are assayed in order to confirm the specific subcellular localization designation, after which an experimentally derived topological map is obtained.

Classically, the locations within an IM protein in which to insert an enzyme fusion or site-specific label were based on the sliding-window hydropathy plot principles developed by Kyte and Doolittle (1982), with primary structure regions of high hydrophobicity suggesting the presence of TMS. More recently, a popular approach to determining the position of reporter fusions (e.g., PhoA, LacZ, GFP) or site-specific labels (e.g., Cys, Met, Asp, Glu residues) within IM proteins has first involved generating a consensus localization of TMS within the proteins through use of numerous in silico topology prediction algorithms (Nilsson et al. 2002). Many laboratories have followed this approach and based on the overlapping positions of predicted TMS, a preliminary in silico-based topology map was first generated, after which targeted reporter fusions or site-specific labels were introduced and detected to validate the proposed model (Fig. 5A). Although TMS prediction algorithms are beneficial for the qualitative identification of integral membrane proteins compared to soluble counterparts, the initial reliance on consensus in silico TMS prediction analyses for designing reporter fusion and site-specific label locations can lead to the exclusion of key characteristics of a protein (Elofsson and Heijne 2007; Islam et al. 2011).

#### Unbiased/randomized reporter and site-specific label positioning

The examination of protein topology in the absence of an “expected” appearance is the ideal manner in which to examine membrane proteins. As such, methods directly involving the protein of interest such as oxidative labeling of native proteins and Nanodisc-assisted DX-MS are advantageous as they do not depend on topology predictions.

Several methods also exist to minimize bias in the use of genetically encoded C-terminal fusions. The use of transposon insertions to create in-frame fusions to reporter proteins is one method to generate nonspecific sites of fusion (Gallagher et al. 2007). However, the tendency of transposons to insert at “hot spots” within a specific gene sequence (Lodge et al. 1988) may limit the random nature of reporter insertion. Alternatively, random exonuclease III-generated and interval-scanning 3′ gene truncation libraries fused to reporter constructs can also be used for this purpose (Islam et al. 2010). As the rate of exonuclease III digestion is known, sequential aliquots from a single digestion reaction can be pooled in a common stop solution, yielding sequentially smaller truncated gene variants which can be re-ligated to the intact reporter gene (Fig. 5B).

## Conclusion: Membrane Proteins – To Topology and Beyond

Although membrane proteins constitute over a quarter of all known proteins (Wallin and Heijne 1998), these cellular machines involved in various assembly, energy production, import/export, and signal transduction events remain poorly characterized relative to their soluble counterparts; this discrepancy is perfectly illustrated by the small fraction of structures in the Protein Data Bank belonging to unique membrane proteins (White 2009). This lack of structural data is due largely to inherent difficulties with overexpression, purification, and crystallization of membrane proteins. Before these difficulties can be overcome by the development of new methods, the use of topological mapping can yield important insights into the properties of various domains such as localization, orientation, and size characteristics.

As such, the main benefit from topology mapping investigations is that they serve as a springboard for comprehensive downstream functional investigations of the respective protein (Islam et al. 2012). Regardless of the method chosen for topological mapping, each with its inherent advantages and drawbacks, simply knowing the number of TMS, or which portions of the protein are exposed, is of no net benefit unless the information gleaned from such results are used to provide a starting point for establishing testable hypotheses based on the interpretation of existing data within a new structural framework.

Ultimately, for some IM bacterial proteins, topological characterization has served to reinforce existing concepts, while for others has provided new insights leading to new avenues of investigation. When used in concert with genetic and biophysical techniques, thorough topological mapping can be a powerful tool in teasing apart the intricacies of membrane protein function. In summary, this review has served to comprehensively and critically analyze the approaches and techniques available to investigators to aid in topological characterization for integral membrane proteins. Information derived from topological mapping can be invaluable in providing credible information with which to form the basis for the design of downstream experiments to shed light on the functional mechanisms of proteins that at one point seemed like black boxes, but for which the tools now exist to extract meaningful and insightful information.
